# Efficient adsorptive removal of hazardous congo red dye using Ce-BTC@microcrystalline cellulose composite

**DOI:** 10.1038/s41598-025-04085-2

**Published:** 2025-06-05

**Authors:** Mostafa A. Sayed, Reda M. Abdelhameed, Ibrahim H. A. Badr, Ali M. Abdel-Aziz

**Affiliations:** 1 Chemistry Department, Faculty of Science, Ain Shams University, Cairo, 11566 Egypt; 2 Applied Organic Chemistry Department, National Research Centre, Dokki, Giza Egypt; 3 Department of Chemistry, Faculty of Science, Galala University, New Galala City, Suez Egypt

**Keywords:** Metal-organic framework (MOF), Ce-BTC@MCC composite, Congo red, Adsorptive removal, Aqueous environment, Environmental chemistry, Materials chemistry, Pollution remediation

## Abstract

In this research, we developed a novel composite material, Ce-BTC@MCC, by combining a metal-organic framework (Ce-BTC) with microcrystalline cellulose (MCC), a recyclable natural product. The surface features of the novel Ce-BTC@MCC composite were carefully investigated through infrared spectroscopy (FTIR), X-ray diffraction (XRD), scanning electron microscopy (SEM), energy-dispersive X-ray spectroscopy (EDX), and N_2_-adsorption/desorption. The ratio of Ce-BTC to MCC in the composite was systematically optimized based on adsorption performance experiments. The developed Ce-BTC@MCC composite significantly outperformed its individual components (Ce-BTC and MCC) in removing Congo Red (CR) dye from water. This enhanced performance is due to the synergistic effect between Ce-BTC and MCC, which enhances the adsorption capacity of the designed composite. A comprehensive investigation was conducted to assess the impact of various parameters, including contact time, pH, temperature, and initial concentration, on the adsorption process. The experimental adsorption data for CR were well-described by the Langmuir isotherm model. The optimized Ce-BTC@MCC composite (20 wt% Ce-BTC content) demonstrated a remarkable maximum adsorption capacity of 926.3 mg/g for CR. The adsorption kinetics followed a pseudo-second-order model (R^2^ = 0.988), and both intraparticle and boundary layer diffusion influenced the rate-limiting step of the adsorption process. A plausible mechanism for the adsorption of CR onto the Ce-BTC@MCC surface was proposed. The results highlight the effectiveness, selectivity, and reusability of the eco-friendly Ce-BTC@MCC adsorbent for removing CR from different real water samples.

**Supplementary Information:**

The online version contains supplementary material available at 10.1038/s41598-025-04085-2.

## Introduction

The contamination of water bodies with synthetic dyes, particularly from textile and industrial wastewater, has become a significant environmental concern^1^. Dye pollution, which is commonly released from industrial activities, can severely degrade water quality. Discharging dye-contaminated wastewater into water bodies disrupts aquatic ecosystems, hampers photosynthesis, and reduces light penetration, affecting the overall aquatic balance^2,3^. Additionally, dyes may contain carcinogenic substances that are harmful to human health. These dyes are persistent and toxic, posing significant environmental and health hazards if untreated. Thus, removing dyes from wastewater is crucial for protecting both aquatic life and human health. Efficient dye removal is essential in water treatment to prevent pollution and meet environmental regulations^4^.

CR, an anionic diazo dye, is one of the most prevalent dyes used in the textile industry, and is known for its high solubility in water and resistance to biodegradation^5^. Its release into the environment poses serious health risks to aquatic life and humans, as it is carcinogenic and can cause severe allergic reactions. The EU and Turkey have prohibited on the use of benzidine-based dyes in textile products^6^. However, owing to their low cost and effectiveness in dyeing, these azo dyes continue to be used in certain regions of Turkey, India, and some third-world countries^7^. Therefore, the removal of CR dye from wastewater is critical for ensuring water quality and ecosystem health.

Diverse techniques, including advanced oxidation, precipitation, solvent extraction, membrane filtering, coagulation-flocculation, photocatalysis, and biodegradation, are used to eliminate potentially hazardous dye pollutants from aqueous environments^8–11^. Among the various methods employed for dye removal, adsorption has emerged as one of the most effective and economically feasible techniques. Adsorptive processes have gained popularity because of their simplicity, efficiency, and ability to treat large volumes of wastewater with minimal chemical input^12,13^. The choice of adsorbent material is a decisive aspect in determining the overall efficiency of the process. Numerous adsorbents, including natural materials, agricultural waste, activated carbon, and novel synthetic materials, have been explored for the adsorption of Congo Red from water^14–17^.

In recent years substantial progress has been made in material science, particularly with the advent of metal-organic frameworks (MOFs). MOFs are a class of porous materials characterized by their organized network structures, which are composed of organic-inorganic hybrids^18–20^. Compared with other nanomaterials, MOFs have attracted significant attention because of their diverse compositions and structures, excellent thermal and mechanical stability, customizable pore characteristics, large surface areas, reproducible metal sites, ease of synthesis, high porosity, and the flexibility to modify their shape and functionality^21–23^. As a result, MOFs have diverse applications across various domains, including gas storage and separation, photochemical processes, catalysis^24^, membranes, sensors, and drug delivery^25–28^. The identification and adsorption of contaminants represent prominent uses of MOFs^29^. MOFs have been utilized as adsorbents for organic pollutants in various applications, including liquid-phase extraction, solid-phase extraction, solid-phase microextraction, preconcentration, and metal ion detection. MOFs can be functionalized to exhibit a net positive or negative charge, facilitating the electrostatic adsorption of anionic or cationic dyes. Additionally, dyes can be adsorbed onto MOFs through physical adsorption, Lewis acid-base interactions, hydrogen bonding, and ion exchange^30,31^.

Cerium-based MOFs have garnered much attention because of their exceptional features in the fields of catalysis and redox chemistry due to their Ce(III)/Ce(IV) redox properties^32,33^. The literature clearly indicates that rare earth metals, such as cerium (Ce), have demonstrated significant potential for removing heavy metals, dyes, pharmaceuticals, and nuclear waste^34,35^. The low-lying 4f orbital of Ce easily forms complexes with organic molecules^36^. Despite being the most abundant rare earth element in the Earth’s crust and widely used in industrial applications, Ce-based materials have rarely been reported for dye adsorption. Conversely, microcrystalline cellulose (MCC), a biopolymer with abundant hydroxyl groups, has garnered attention due to its renewability, biocompatibility, and inherent porous structure, making it a promising candidate for dye adsorption^37,38^. However, despite its favorable characteristics, MCC suffers from limited adsorption capacity and selectivity, which hinders its efficiency in large-scale applications. In addition, the adsorption capacity of bare MCC is too low for practical use in wastewater treatment operations^39^.

In recent years, the integration of Metal-Organic Frameworks (MOFs) with MCC has emerged as a promising strategy to overcome these limitations^40,41^. MOFs, with their high surface area, tunable porosity, and functionalized metal sites, significantly increase the adsorption efficiency of MCC, especially for the removal of organic pollutants such as dyes^42^. The synergistic effect between the porous structure of MCC and the metal coordination sites of MOFs allows for improved adsorption capacity, stability, and reusability, making this hybrid material highly attractive for environmental remediation^41,43^. MCC-based composites have shown effective adsorption performance for water-polluting dyes, exhibiting a uniform adsorption site distribution and forming a monolayer of adsorbate^44^. Consequently, integrating MCC with other adsorbents in composite formulations may enhance its physical features and produce adsorbents that are more potent^41,44,45^. By exploring the influence of MOF incorporation on MCC in both single- and multi-component adsorption systems, we can gain deeper insights into the material’s performance under varying conditions, offering a pathway toward the design of more efficient adsorbents for dye removal from industrial effluents^46,47^. Therefore, the formulation of a composite based on the Ce-BTC framework and MCC could significantly improve, through synergistic effects, the properties of the composite in comparison with those of its constituents. Furthermore, the development of a new and multifunctional composite material, such as Ce-BTC@MCC, with dual functionalities underscores the efficiency of a unified solution.

In this study, a novel adsorbent composed of a Ce-BTC metal-organic framework and MCC was synthesized in situ for the adsorption-based removal of harmful Congo red (CR) dye. The Ce-BTC@MCC composite was fully characterized through surface analysis and spectroscopic techniques. Various experimental factors, including contact time, pH, and preliminary dye concentration were evaluated. A comprehensive investigation of the isotherms and kinetics was conducted, and a potential adsorption mechanism was proposed. This investigation makes a significant contribution to the field of sustainable and efficient wastewater treatment strategies to address dye pollution.

## Experimental

### Materials

The chemicals employed in this study were of analytical grade and were used as received. Absolute ethanol (EtOH), cerium nitrate hexahydrate (Ce(NO_3_)_3_.6H_2_O), 1,3,5-benzenetricarboxylic acid (BTC), and microcrystalline cellulose were procured from Merck (Darmstadt, Germany). Congo red (CR) dye was procured from (LOBA, India) and used without further purification.

### Preparation of the Ce-BTC MOF

Ce-BTC was formulated by dissolving 0.42 g of 1,3,5-benzenetricarboxylic acid in 100 mL of DMF. At the same time, 10 mL of DMF was used to dissolve Ce(NO_3_)_3_. The two solutions were combined and stirred for 20 min at 25 °C. For 24 h, the reaction mixture was maintained at 100 °C in an oven. After this time frame, the Ce-BTC powder was collected, rinsed with pure EtOH, and then filtered through Whatman filter paper. After the sample was dried, it was finally ground into a white solid, which was then used in the following experiments.

### Preparation of the Ce-BTC@MCC composite

The Ce-BTC@MCC composite was formulated by immersing 0.5 g of MCCs into 50 mL of DMF containing 0.316 g of Ce(NO_3_)_3_ and stirring for 1 h at room temperature. The mixture was then continuously stirred with 50 mL of DMF containing 0.42 g of 1,3,5-benzenetricarboxylic acid. Two mL of triethylamine was added to the above mixture, and the mixture was subsequently incubated for 8 h at 100 °C. After the white precipitate formed and naturally cooled to room temperature, the sample was centrifuged to filter, and then centrifuged again with ethanol and DMF, and then dried under vacuum for 12 h at 80 °C to generate the Ce-BTC@MCC composite.

**Scheme 1:**
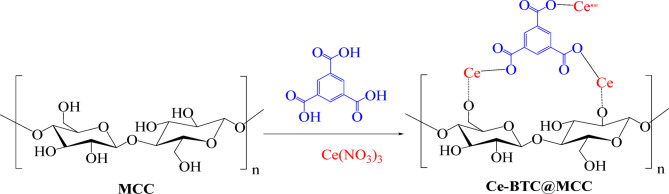
The formation mechanism of the Ce-BTC@MCC composite.

Following the steps shown in Scheme 1, both the Ce-BTC and Ce-BTC@MCC hybrids were formulated via the conventional solution method, which revealed the structural composition of the Ce-BTC@MCC composite. Ce-BTC was prepared in suite while MCC was present. When the synthesized Ce-BTC@MCC composite was formed, the compositions of Ce-BTC and MCC changed. FTIR and XRD were used to confirm the changes in the bonding and structural features of the developed sorbents.

### Batch adsorption experiments

#### Effect of pH

The influence of pH on the adsorption of CR onto Ce-BTC@MCC was examined by combining 0.02 g of MOF powder with 50 mL of dye solution at an initial concentration of 100 mg/L across various pH values (3.0–11.0) at ambient temperature. The pH was modified via 0.1 M NaOH and 0.1 M HCl solutions and assessed with a pH meter. The mixture was stirred for 30 min at a steady speed of 250 rpm. The concentrations of the dyes were assessed via a double beam UV–VIS spectrophotometer. Calibration curves were generated via standard CR solutions prior to measurement.

#### Surface analysis

The method published by Dahri et al.^48^ was used to calculate the point of zero charge (pH_PZC_) of the synthesized adsorbent. The experiment was conducted using a set of 100 mL Erlenmeyer flasks, each containing 25 mL of KNO_3_ solution with a concentration of 0.1 M. Additionally, 0.02 g of adsorbent powder was added to each flask. Solutions containing 0.1 M NaOH and 0.1 M HCl were employed to modify the pH of the KNO_3_ solutions within the range of 2.0–12.0. Afterward, the KNO_3_ solutions were stirred vigorously for 24 h at 25 °C, and the final pH was determined. The pH_PZC_ was determined by graphing the difference in pH (ΔpH) against the initial pH.

#### Equilibrium and kinetic studies

Batch adsorption experiments were conducted by adding 0.02 g of sorbent to 50 mL of dye solution with initial concentrations ranging from 20 to 3000 mg/L. The mixtures were agitated at 250 rpm for 30 min, and the residual dye concentration was measured via UV-VIS spectrophotometry. All the adsorption experiments were carried out three times (*n* = 3), the mean values are reported and the error bars included in each graph represent the corresponding standard deviations. The removal efficiency of the Ce-BTC@MCC adsorbent can be estimated via Eq. 1:

1 $$\text{Removal}, {\%}=\:\frac{{\text{C}}_{0}-{\text{C}}_{\text{t}}}{{\text{C}}_{0}}\times\:100$$

where $\:{\text{C}}_{0}$ and $\:{\text{C}}_{\text{t}}$ (mg/L) are the initial and proceeding concentrations of the adsorbate (CR), respectively. The adsorption capacity, Qe (mg/g), was calculated at equilibrium via Eq. 2:

2 $$\:{\text{Q}}_{\text{e}}=\frac{{(\text{C}}_{0}-{\text{C}}_{\text{e}})\text{V}}{\text{W}}$$

where C_0_ (mg/L) and Ce (mg/L) represent the concentrations of the CR dye at the beginning and at equilibrium, respectively. The volume of the solution is V (L), and the mass of the dry sorbent (W) used is expressed in grams. Kinetic experiments were conducted similarly to the equilibrium tests. Samples were taken at specific time intervals, and the amount of sorption, Qt, was determined via Eq. 3:

3 $$\:{\text{Q}}_{\text{t}}=\frac{{(\text{C}}_{0}-{\text{C}}_{\text{t}})\text{V}}{\text{W}}$$

where C_t_ (mg/L) represents the liquid-phase concentration of dye at any time.

## Results and discussion

### Characterization of sorbents

#### X-ray diffraction

**Fig. 1:**
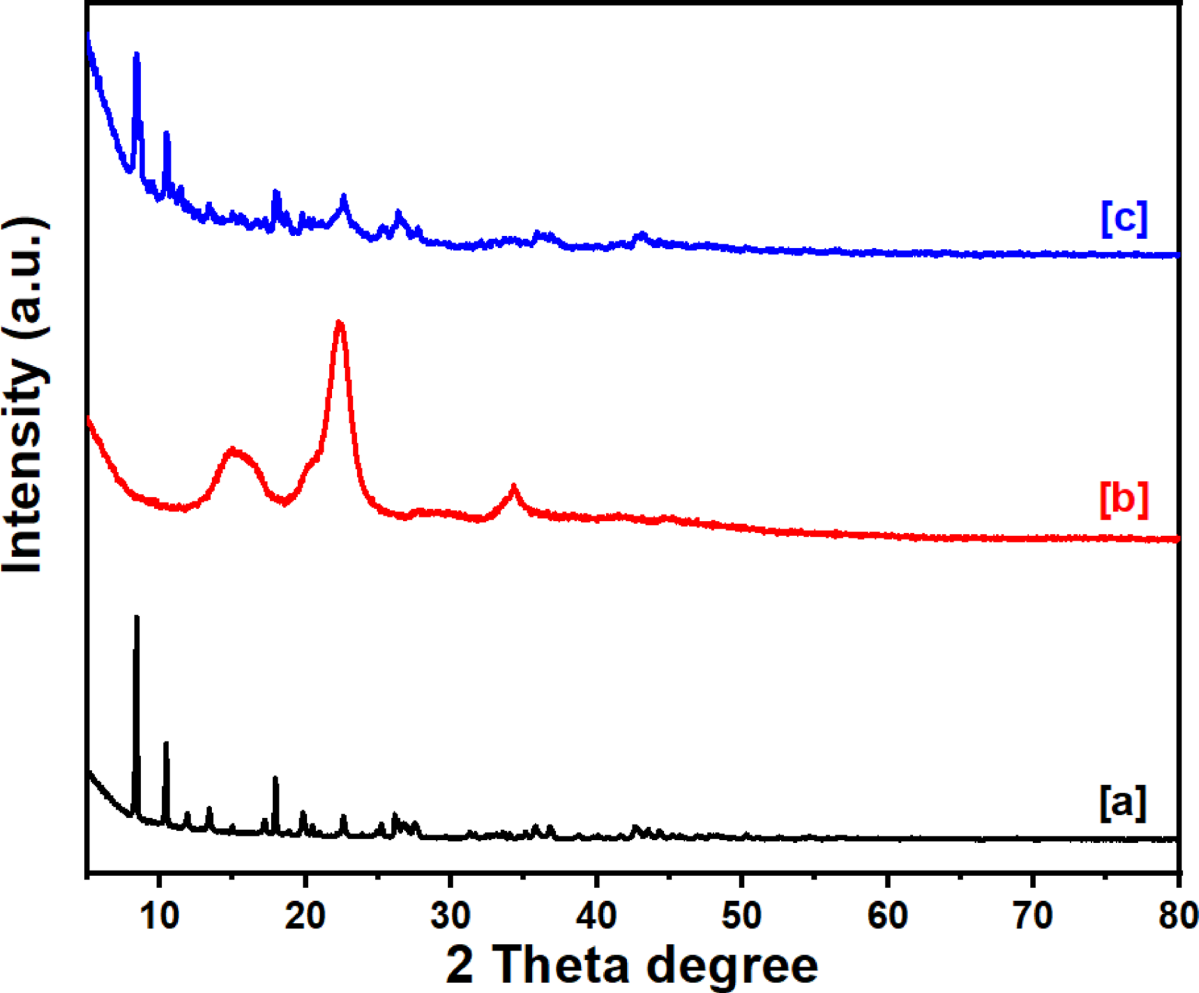
PXRD patterns of [a] Ce-BTC, [b] MCC, and [c] Ce-BTC@MCC composite.

The diffraction pattern of Ce-BTC is shown in Fig. 1a. The unit cell characteristics of the PXRD peaks were as follows: α = β = γ = 90° (rhombohedral), a = 10.9375, b = 6.7299, and c = 18.584. The space group Pnm a 63 was used to index the PXRD peaks. The large peaks at 12.1°, 20.1°, 22.3°, and 34.6° which are characteristic of cellulose II crystals were visible in the MCC crystal structures shown in Fig. 1b, which displays the MCC diffraction pattern. The diffraction patterns of Ce-BTC@MCC are shown in Fig. 1c. The diffraction bands of Ce-BTC were clearly visible in the composite, indicating that MCC successfully formed crystalline Ce-BTC^49^. The XRD patterns of the Ce-BTC@MCC composite are shown in Fig. 1c. The characteristic peaks of Ce-BTC were observed at 8.2 and 12.1°. In addition, the XRD pattern of Ce-BTC@MCC shows a noticeable peak in the 2θ range between 20 and 30°, which is normal in cellulose. These findings confirm the crystallinity and purity of the formulated composites^50,51^.

#### Infrared spectroscopy

**Fig. 2:**
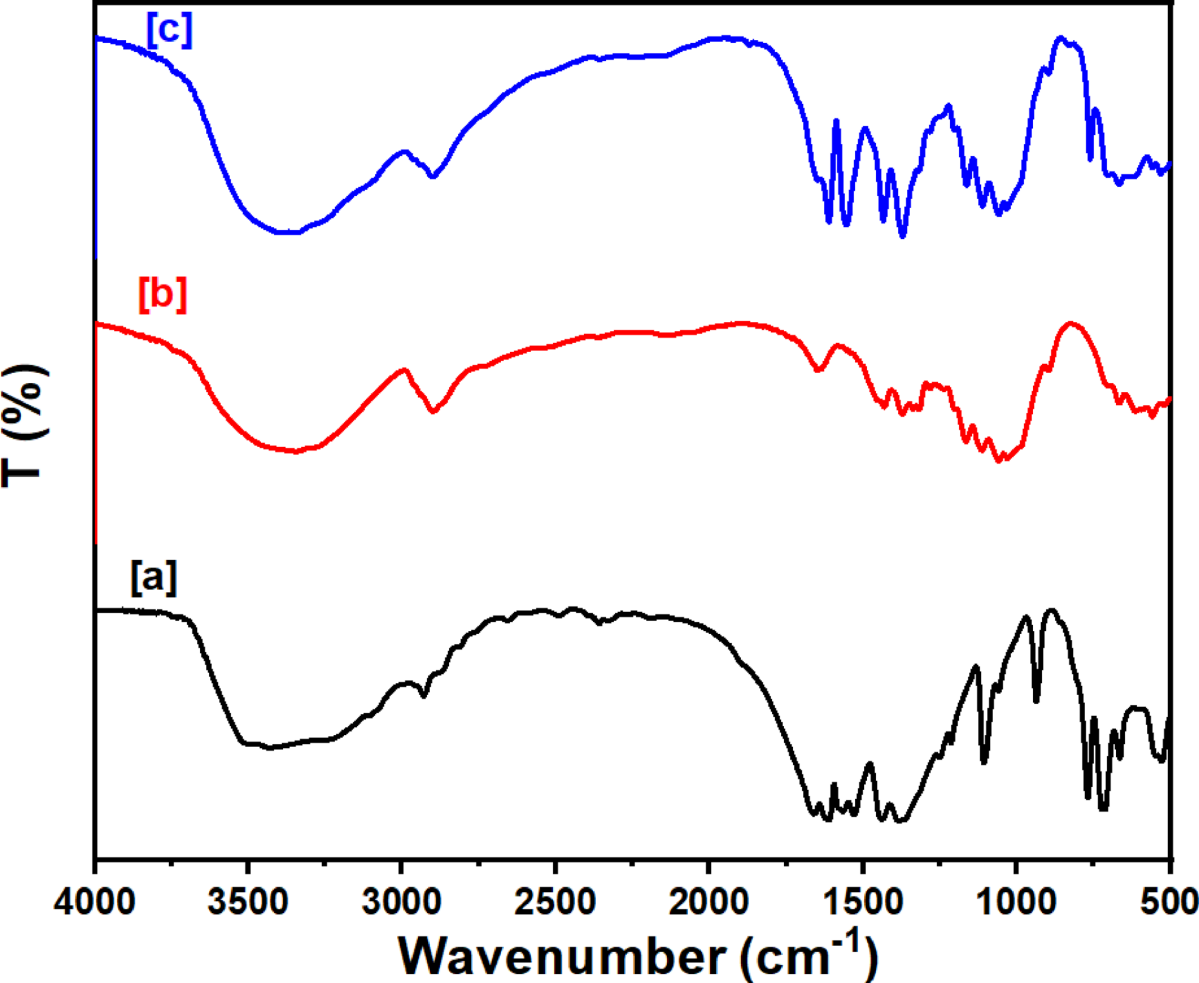
FTIR spectra of [a] Ce-BTC, [b] MCC, and [c] the Ce-BTC@MCC composite.

The FT-IR spectra of Ce-BTC, presented in Fig. 2a, exhibit characteristic peaks at 3306 cm^−1^, corresponding to O-H stretching via hydrogen bonds with water, and peaks at 1149 cm^−1^ and 1021 cm^−1^, indicative of Ce-O bonds^49^. The spectral feature corresponding to the bending mode of free water is present at 1632.1 cm^−1^^52–54^. Carboxylic acid groups exhibit symmetric and asymmetric O-C-O stretching within the 1700–1300 cm^−1^ range^55^. The asymmetric O-C-O stretching is associated with the peaks at 1574.5 cm^−1^, 1556 cm^−1^, and 1510 cm^−1^, whereas the symmetric O-C-O stretching is associated with the peaks at 1435.5 cm^−1^ and 1392 cm^−1^^56^. The FTIR spectrum of MCC, displayed in Fig. 2b, reveal bands at 1024 cm^−1^ (C-O), 2900 cm^−1^ (CH_2_-CH), and 3347 cm^−1^ (O-H)^57^. The FTIR spectrum of the composite, presented in Fig. 2c, reveals absorption bands characteristic of both Ce-BTC and MCC, demonstrating successful composite formation^41^.

#### SEM/EDX analysis

**Fig. 3:**
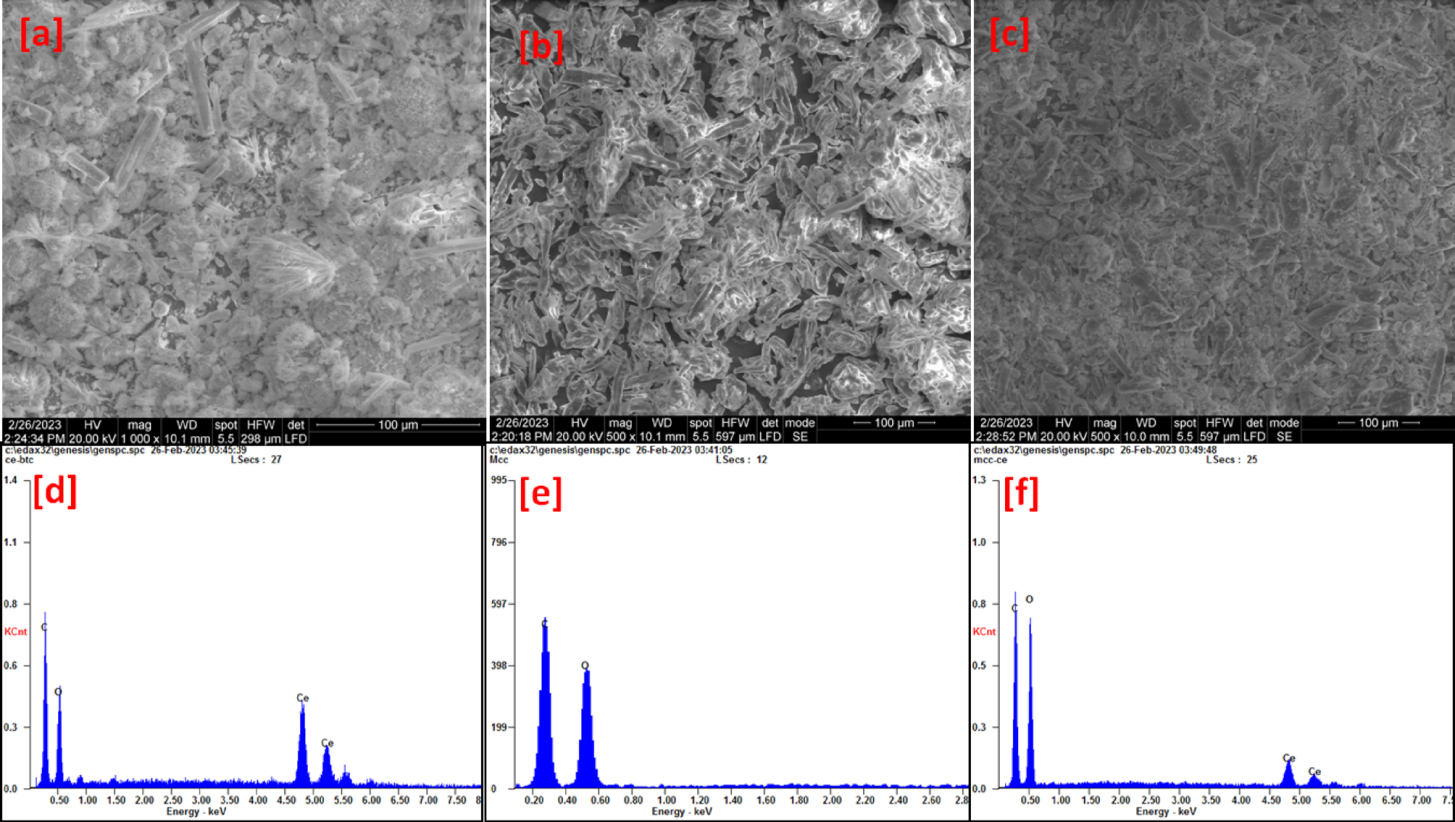
SEM images of [a] Ce-BTC, [b] MCC, and [c] Ce-BTC@MCC, and EDX images of [d] Ce-BTC, [e] MCC, and [f] Ce-BTC@MCC.

Figure 3a shows the typical crystal morphology of Ce-BTC in the FE-SEM images. For comparison, the morphology of MCC was examined. The SEM image in Fig. 3b shows a 3D network structure with uniformly distributed particles and massive particle clusters. The morphological characteristics of Ce-BTC@MCC were investigated via SEM, and the results are shown in Fig. 3c. The crystal size of Ce-BTC@MCC was measured under a microscope and was found to be 12.1 × 2.2 mm. The crystal structure feature was quite different from that of MCC and Ce-BTC, indicating that the two compounds were successfully encapsulated. EDX analysis revealed the presence of oxygen, carbon, and cerium ions in Ce-BTC (Fig. 3d), whereas Fig. 3e shows that only carbon and oxygen were related to MCC. Figure 3f shows the carbon, oxygen, and cerium contents of Ce-BTC@MCC, confirming the chemical formula of the target composite.

#### XPS analysis

To investigate the surface composition, oxidation states, and binding energies of Ce-BTC@MCC, XPS analysis was performed, as presented in Fig. S1. The presence of C 1s, O 1s, and Ce 3d elements was confirmed by the Ce-BTC@MCC survey. For a more comprehensive understanding, high-resolution spectra are also thought to be important. Three separate peaks representing C-C, C-O-C, and C-C = O were visible in the C1s region at 283.3, 284.7, and 287.5 eV, respectively. The oxygen component of the carboxyl groups in the ligand is responsible for the two peaks at binding energies of 530.4 and 528.3 eV which represent C = O and C-O, respectively, and fits the core level spectrum of O 1s, as shown in Fig. S1. The presence of redox-active cerium atoms in the framework is justified by the core level spectrum of Ce 3d, which shows a mixed valence state (Ce^3+^/Ce^4+^) in Ce-BTC@MCC. Ce^4+^ is responsible for the peaks with centers at 881.8, 899.5, and 915.8 eV, whereas Ce^3+^ peaks are known for their peak areas at 886.8 and 905.2 eV. Following the immobilization process, the spectrum of C 1s related to C-O showed a small shift compared with that of the parent compound. Similarly, the O 1s spectrum related to O-H showed a very small shift compared to that of the parent compound, enhancing the ability of material to interact with the cellulose matrix.

XPS spectra of Ce-BTC@MCC were also taken after adsorption to better investigate the adsorption mechanism (Fig. S2). Following the adsorption process, the Ce-BTC@MCC elements were visible in the XPS scan, as shown in Fig. S2. Four peaks can be identified in the C 1s spectra, and they are attributed to π-π*, O = C-O, C-O, and C-C/C-H. The negative shift of 0.23 eV following adsorption suggests a robust hydrogen bond-based chemical interaction between the dye species and C-C/C-H. The π-π* component was identified as the satellite peak at 290.1 eV, and following dye adsorption, its proportion decreased from 6.16 to 2.15%, suggesting that the adsorbent and the dye species interacted with π anions. The two peaks in the N1 spectrum at 397.4 eV are attributed to C–N bonds, whereas the -NH_2_ groups in Ce-BTC@MCC and the coordination between the nitrogen atoms of the -NH_2_ and Ce–O groups are responsible for 399.6 eV. Ce-oxides, -OH, C = O, and C–O/CO_3_^2−^ are represented by the four distinct peaks in the O1 spectrum, which are located at 529.6, 531.3, 530.7, and 533.5 eV. It is evident that Ce 3d displayed broad peaks, suggesting that Ce exists in a variety of oxidation states. The spin-orbit dimers of Ce 3d_5/2_ and Ce 3d_3/2_ were fitted to the Ce 3d plot. The moon peaks of Ce^4+^ 3d_5/2_ and Ce^4+^ 3d_3/2_ are responsible for the peaks at 885.4 eV and 906.5 eV, respectively. These peaks most likely originate from the Ce–O bond in Ce-BTC@MCC.

#### BET analysis

**Fig. 4:**
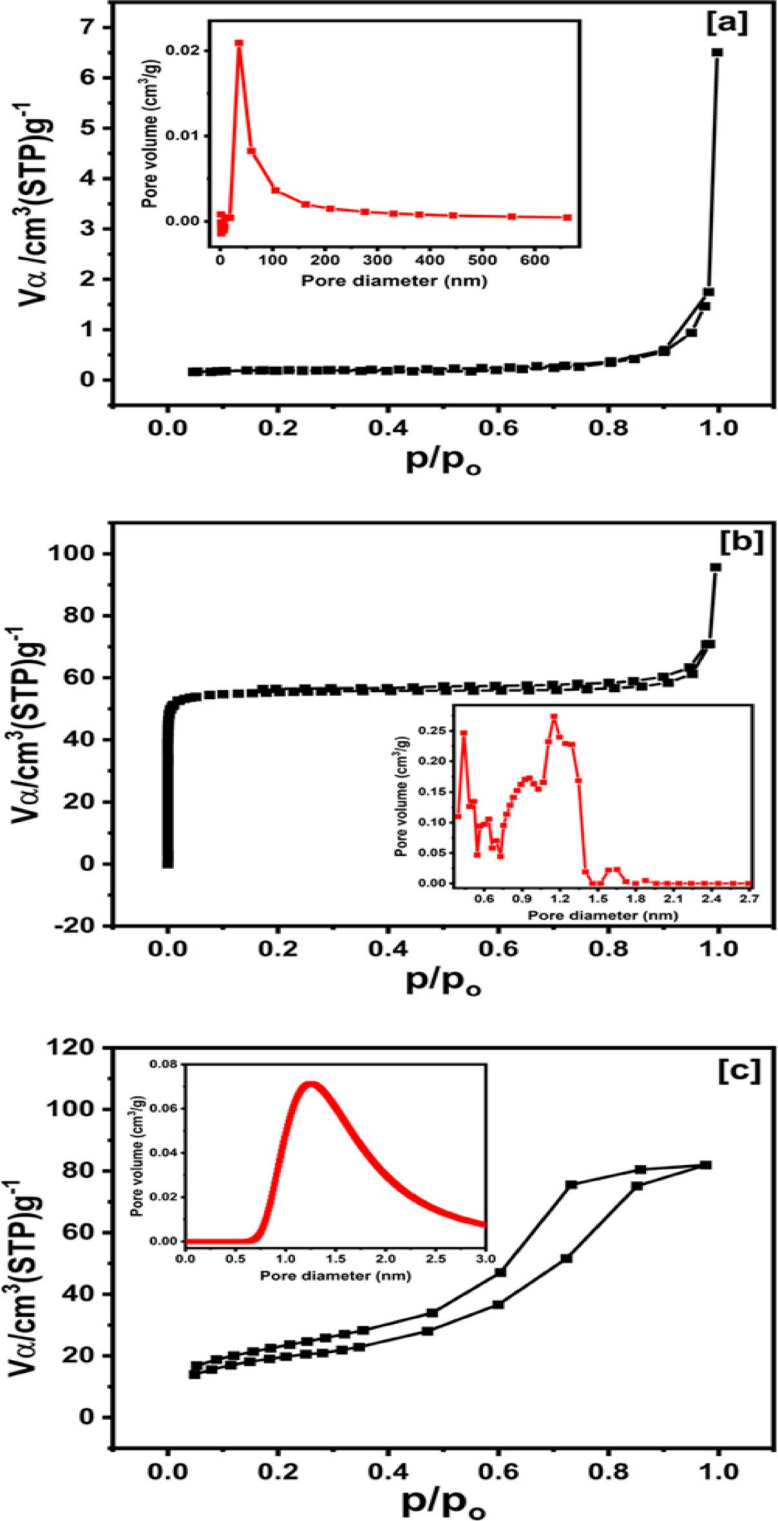
N_2_ adsorption/desorption isotherms with pore size distributions (PSDs; inset) for: [a] MCC, [b] Ce-BTC, and [c] the 20 wt% Ce-BTC@MCC composite.

The N_2_ adsorption-desorption isotherms for MCC, Ce-BTC, and 20 wt% Ce-BTC@MCC are depicted in Fig. 4. These isotherms, classified as type I, indicate microporous structures, with corresponding surface areas of 0.713, 221, and 67.9 m^2^g^−1^. Through N_2_ adsorption experiments, the differences in the porosity properties of the Ce-BTC and Ce-BTC@MCC composites were investigated. The adsorption curves of the Ce-BTC and Ce-BTC@MCC composites are believed to most likely fall within the first-type adsorption curve, with hysteresis loops falling within the H3 and H1 categories, according to Brunauer’s classification of the five types of adsorption curves. At low pressure, the adsorption curve is convex, indicating a strong interaction between the adsorbent and the adsorbent. Ce-BTC and Ce-BTC@MCC composites are classified as mesoporous by the International Union of Pure and Applied Chemistry (IUPAC) because the width of the mesopores ranges from 2 to 50 nm. The pore size distributions of MCC, Ce-BTC, and Ce-BTC@MCC are shown in the inset of Fig. 4, which further confirms the change in pore size. The average pore sizes of MCC, Ce-BTC, and Ce-BTC@MCC were approximately 86.5 nm, 1.14 nm, and 104.5 nm, respectively. The incorporation of Ce-BTC into the composite increased the pore volume, likely due to dispersion of Ce-BTC on the composite surface^58^. MOFs such as Ce-BTC typically have highly porous structures, so incorporating them could increase the total pore volume, especially if the Ce-BTC does not fill in the pores of the composite but is dispersed in a way that creates additional void spaces^59,60^. Additionally, the nitrogen adsorption–desorption isotherms and corresponding pore size distribution analyses provide significant insights into the influence of Ce-BTC loading on the textural properties of Ce-BTC@MCC composites. The sample with 5 wt% Ce-BTC loading (Fig. S3a) displays relatively low nitrogen uptake (~ 35 cm³/g at P/P⁰ ≈ 1.0), corresponding to a low specific surface area of approximately 6 m²/g. Its broad pore size distribution, extending to ~ 250 nm, is indicative of a poorly defined macroporous structure, likely resulting from insufficient framework formation and non-uniform pore development at low Ce-BTC content. In contrast, the 20 wt% Ce-BTC@MCC composite (Fig. S3b) exhibits the highest nitrogen uptake (~ 90 cm³/g), with a well-defined microporous structure centered at ~ 1.5 nm and a significantly higher surface area of ~ 68 m²/g. This suggests optimal dispersion of the Ce-BTC component within the MCC matrix, yielding a highly porous and uniform framework. However, further increasing the Ce-BTC content to 25 wt% (Fig. S3c) leads to a notable decline in nitrogen uptake (~ 45 cm³/g) and surface area (~ 12 m²/g). The corresponding pore size distribution shows a broad peak in the 80–100 nm range, reflecting the emergence of larger meso- and macropores due to particle aggregation. These findings strongly support the conclusion that excessive Ce-BTC loading results in coagulation and pore blockage, ultimately diminishing the accessible surface area and overall porosity of the composite.

Although the surface area of the Ce-BTC@MCC composite is lower than that of the Ce-BTC MOF, the optimized composite contributes to improved adsorption performance with higher adsorption capacity compared to the individual components (MCC and Ce-BTC). Numerous studies reported high adsorption capacities despite low surface areas^61^. The surface area is unlikely to be the sole or primary factor driving the significant adsorption behavior of Ce-BTC@MCC towards CR dye. Importantly, enhanced adsorption is also due to other intrinsic properties of the composite. Notably, the functional groups within the Ce-BTC@MCC matrix create strong interaction sites, including hydrogen bonding, π-π interactions, and coordination with CR molecules, significantly improving CR adsorption. These interactions enhance the adsorption of CR on the mesoporous composite. These combined factors (hydrogen bonding, chelation, π-π stacking interactions) are plausibly responsible for the superior adsorption behavior of Ce-BTC@MCC, making it an effective adsorbent for CR dye (as discussed below in the adsorption mechanism, Sect. 3.7, which was confirmed by XPS analysis in Sect. 3.1.4).

### Factors affecting adsorption

#### Type of adsorbent

**Fig. 5:**
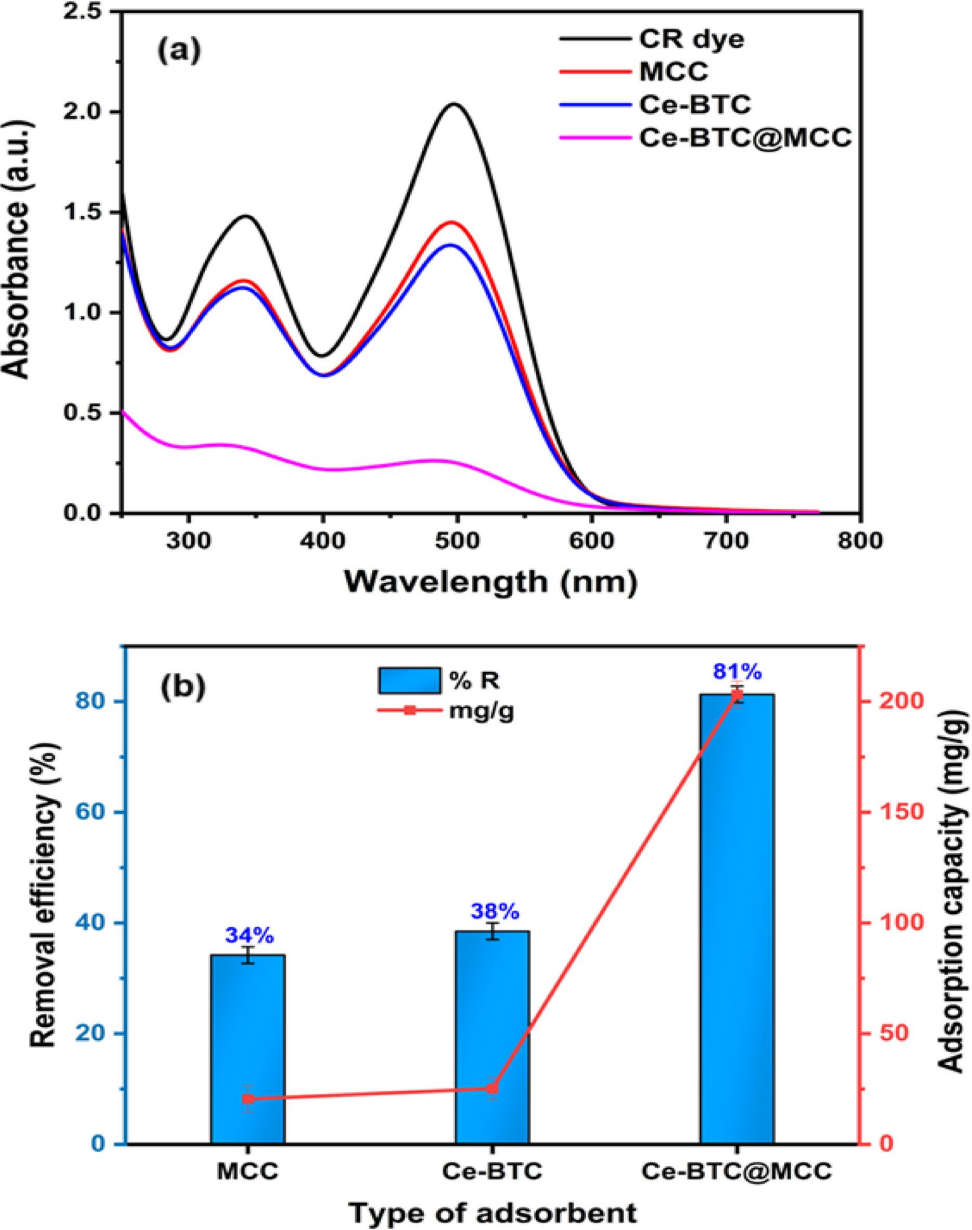
**(a)** Absorption spectra of CR dye in the presence of different adsorbent species and **(b)** the adsorption efficiency of the adsorbent species (experimental conditions: pH = 5.0, contact time = 30 min, temperature = 298 K, concentration of CR = 100 mg/L, and adsorbent dose = 0.4 g/L).

An anionic organic dye pollutant, Congo red (CR), was selected to investigate the adsorption efficiency of the Ce-BTC@MCC composite. The adsorption capacities of the pristine components of the prepared composite, namely, Ce-BTC and MCC for CR were 25 and 20 mg/g, respectively. However, the Ce-BTC@MCC composite had an adsorption capacity of 203 mg/g for CR when the original concentration was 100 mg/L as presented in Fig. 5. The results demonstrated that the integration of Ce-BTC with MCC significantly enhanced its ability to adsorb CR dye through synergistic interactions^41,62^. The ratio of Ce-BTC to MCC in the composite was systematically optimized on the basis of adsorption performance experiments. Initially, we observed that increasing the amount of Ce-BTC in the composite led to an improvement in the adsorption capacity, as Ce-BTC contributes significantly to the number of adsorption sites because of its high surface area and porosity. However, after reaching an optimal ratio of 20 wt% Ce-BTC in the Ce-BTC@MCC composite, further increases in the Ce-BTC content resulted in a decrease in the adsorption efficiency. This decrease is likely due to the agglomeration of Ce-BTC at higher concentrations, which can reduce the accessible surface area and number of adsorption sites, thus limiting the overall adsorption capacity. The optimal 20 wt% Ce-BTC content was determined through a series of adsorption experiments, where the adsorption capacities were measured at varying Ce-BTC loadings (Fig. S4). We observed that at this ratio, the composite exhibited the highest adsorption efficiency, striking a balance between sufficient Ce-BTC content for adsorption and maintaining effective dispersion in the MCC matrix. Furthermore, the Ce-BTC@MCC (20 wt% Ce-BTC content) composite was chosen for additional studies aimed at optimizing the adsorption properties.

#### Effect of pH

**Fig. 6:**
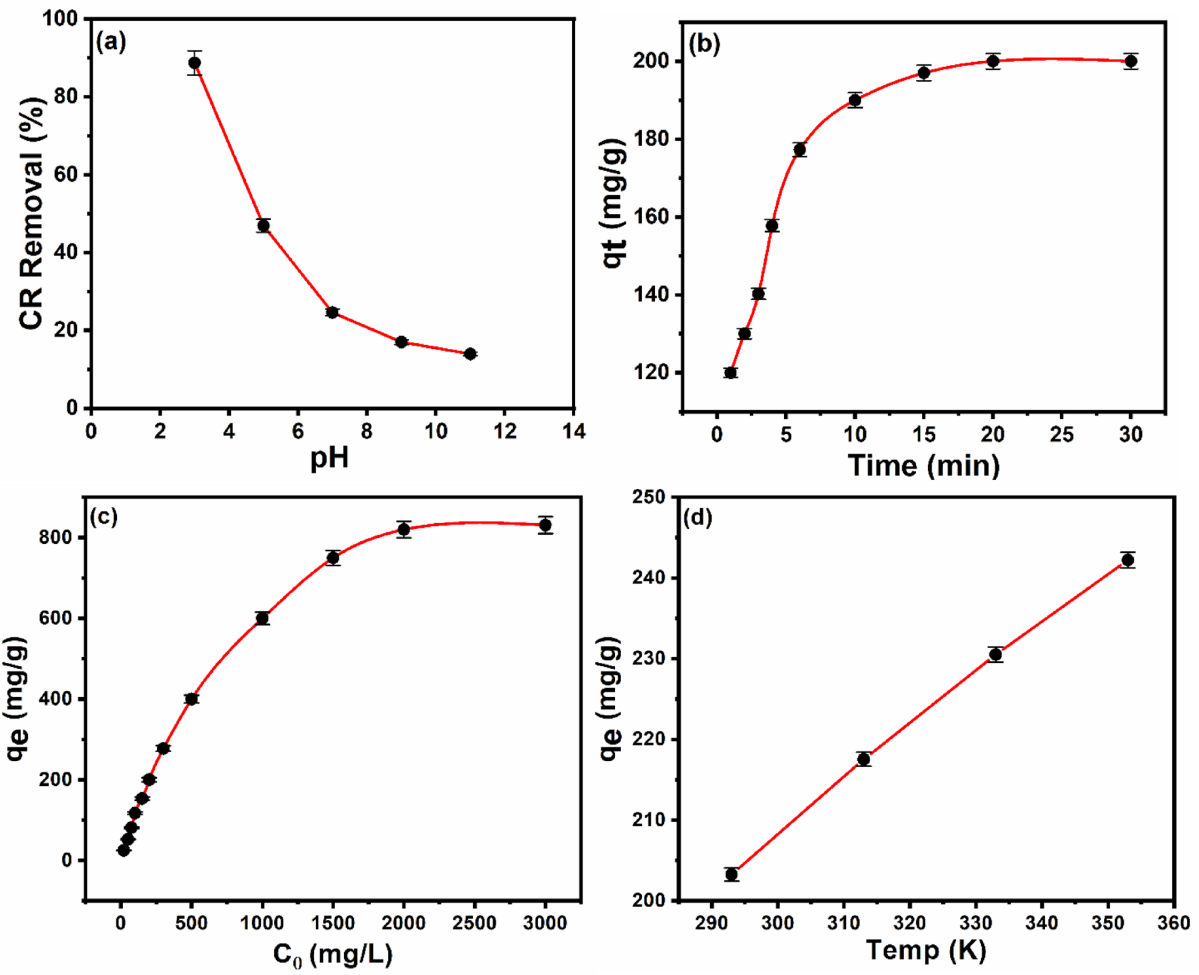
(a) Effect of the solution pH (contact time = 30 min, temperature = 298 K, and concentration of CR = 100 mg/L). (b) Effect of contact time (pH = 5.0, temperature = 298 K, concentration of CR = 100 mg/L). (c) Effect of initial concentration (pH = 5.0, contact time = 30 min, temperature = 298 K). (d) Effect of temperature (pH = 5.0, contact time = 30 min, concentration of CR = 100 mg/L) on the adsorption of CR dye onto the Ce-BTC@MCC composite (adsorbent dose = 0.4 g/L).

Indeed, the pH of the solution is essential in defining the surface characteristics of the adsorbent and the level of ionization of the adsorbate. This eventually effects on the adsorptive removal efficiency^63^. The impact of pH on the ability of Ce-BTC@MCC to remove CR dye was scrutinized over the pH range of 2–11 for an initial dye concentration of 100 mg/L as presented in Fig. 6 (a). In this investigation, increasing the pH from 3 to 11 resulted in a decrease in the CR removal efficiency from 98 to 13%, respectively. Typically, the adsorption of anionic dyes, such as Congo red, decreases with increasing pH on the basis of electrostatic interactions between the dye molecules and the adsorbent species^64,65^. To provide an explanation for this behavior, the point of zero charge (pHpzc) of Ce-BTC@MCC was determined and observed at ~ 3 as shown in Fig. S5. Consequently, at pH = 3, the neutral nature of the adsorbent surface creates more available binding sites, enhancing the accessibility of CR dye molecules for adsorption^66^. At elevated pH values (pH > pH_pzc_), the negatively charged surface of the adsorbent repels anionic dye molecules due to electrostatic repulsion forces, decreasing the adsorption of CR molecules onto the surface^67^. Additionally, the lower adsorption of CR dye at alkaline pH is attributed to the excess OH⁻ ions, which compete with the dye anions for available adsorption sites^68^. Therefore, the effect of pH suggested that electrostatic interactions significantly impacted the adsorption processes of CR dye by the Ce-BTC@MCC composite. Moreover, as the pH decreases (pH < 5), the color of the CR solution changes from red to dark blue due to conformation changes^69^. Therefore, pH 5 was selected as the optimal level for further studies, as it is also near the natural pH suitable for environmental applications.

#### Effect of contact time

An investigation was conducted to examine the influence of contact time on the efficacy of adsorption to determine the rate and duration needed to achieve equilibrium. The effect of contact time on the removal efficiency of CR dye using the Ce-BTC@MCC composite is presented in Fig. 6 (b). The ability of Ce-BTC@MCC to adsorb increased as the contact time increased. The findings indicated that the adsorption process occurred rapidly, with most of the dye being absorbed within the initial 10 min, whereas equilibrium was reached in 15 min when the Ce-BTC@MCC adsorbent was used. Interestingly, approximately 99% of the removal was accomplished during the first 15 min of the experiment. The initial increase in adsorption was caused by the accessibility of free adsorption sites on the adsorbent surface, which gradually became saturated as the dye molecules attached to the adsorbent^70^. This finding is consistent with those of previous studies^71,72^.

#### Effect of the initial concentration

As the number of adsorbate molecules increases, the likelihood of adsorbate-adsorbent interactions increases, leading to a greater level of adsorption^73^. The initial adsorbate concentration influences the resistance to mass transfer between the adsorbate and the solution phase. There is always some resistance to mass transfer from the liquid phase to the solid phase. This resistance is inversely related to the concentration difference. As a result, an increase in the adsorbate concentration in the liquid phase acts as a driving force to reduce the mass transfer resistance^74^. The impact of the starting concentration of CR dye on the overall efficacy of the Ce-BTC@MCC adsorbent was assessed within the range of 20–3000 mg L^−1^. As shown in Fig. 6 (c), the quantity of adsorbed CR at equilibrium rose linearly and notably increased the CR concentration. The extent of CR adsorption increased from 25 mg/g to 750 mg/g as the initial concentration rose from 20 mg/L to 1500 mg/L, respectively, with a maximum adsorption capacity of 834.5 mg/g observed at an initial concentration of 2500 mg/L. The observed effect of the initial adsorbate concentration can be explained by the idea that higher initial concentrations enhance the driving force toward the surface of the Ce-BTC@MCC sorbent, leading to increased Qe values. However, when the adsorbate concentration exceeds the optimal level, the number of offered adsorption sites decreases, causing a reduction in removal efficiency^75^.

#### Effect of temperature

The impact of temperatures ranging from 293 K to 353 K on the adsorption efficiency of Ce-BTC@MCC was examined (see Fig. 6 (d)). When the temperature increased from 20 °C (293 K) to 80 °C (353 K), the adsorption efficiency for CR increased. This enhancement in efficiency can be attributed to the improved diffusion rate of dye molecules through both the external boundary layer and internal pores of the Ce-BTC@MCC particles^76^. Moreover, varying the temperature affects the adsorbent’s equilibrium capacity for a given adsorbate^77,78^. Therefore, the adsorption process of CR seems to be endothermic, as the sorption capacity increases with increasing temperature. This finding is consistent with observations reported in various studies in the literature^79,80^.

### Adsorption isotherms

**Fig. 7:**
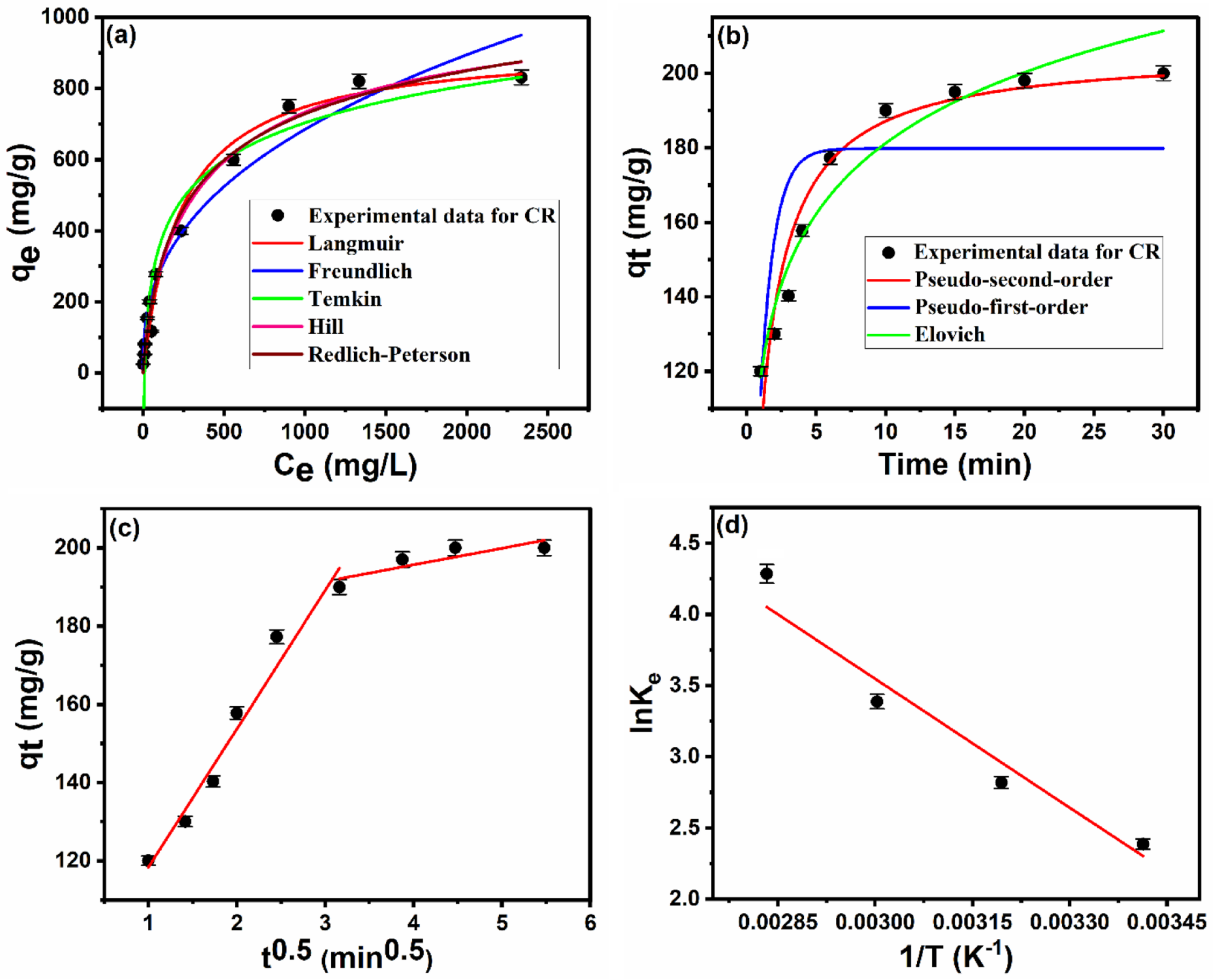
(a) Nonlinear fitting plots of the Langmuir, Freundlich, Temkin, Redlich-Peterson, and Hill adsorption isotherm models of CR on Ce-BTC@MCC. (b) Nonlinear fitting plots of pseudo-first-order, pseudo-second order, and Elovich kinetic models for the adsorption of CR on Ce-BTC@MCC. (c) Intraparticle diffusion for the adsorption of CR dye on the Ce-BTC@MCC composite. (d) The thermodynamic parameters were calculated by plotting lnKe versus 1/T. The experimental conditions are similar to those in Fig. 6.

To gain insight into the nature of the interaction between CR and Ce-BTC@MCC, the equilibrium adsorption data were fitted to various isotherm models, including the Langmuir (Eq. (S1))^81^, Freundlich (Eq. (S2))^82^, Temkin (Eq. (S3))^83^, Redlich-Peterson (Eq. (S4))^84^, and Hill (Eq. (S5))^85^ models **(**Fig. 7a**);** subsequently, the parameters evaluated from these models are presented in Table 1. The Langmuir isotherm model, with a correlation coefficient of R² = 0.9849, provided the best fit to the experimental data. The maximum adsorption capacity (q_m_) of Ce-BTC@MCC for CR, as determined from the Langmuir model, was 926.3 mg/g (see Table 1). The Hall separation factor (R_L_), a dimensionless constant, is a useful parameter for characterizing the nature of the Langmuir isotherm, as defined by Eq. (4)^86^,

4 $$\:{\text{R}}_{\text{L}}=\:\frac{1}{(1+\:{\text{K}}_{\text{L}\:\:}{\text{C}}_{0\:})}$$

where C_0_ (mg/L) is the highest initial adsorbate concentration, while K_L_ (L/mg) is the Langmuir constant. The value of R_L_ provides insights into the nature of the adsorption process to be favorable (0 < R_L_ < 1), unfavorable (R_L_ > 1), linear (R_L_ = 1), or irreversible (R_L_ = 0)^86^. The R_L_ value for the adsorption of CR onto Ce-BTC@MCC was 0.073, which is close to zero confirming that the adsorption of CR onto Ce-BTC@MCC is favorable and irreversible.

The Freundlich isotherm was also applied to the experimental data (Table 1). The value of **n**, which is greater than unity (*n* = 2.6), indicates a favorable adsorption process^87–89^, which is consistent with the R_L_ value. A comparative analysis of the correlation coefficients (R²) and chi-square (χ²) values of the five studied isotherms revealed that the Langmuir isotherm model provided the best fit to the experimental data. The highest R² value of 0.9849 and lowest χ² value of 1909.2 for the Langmuir model indicate that the adsorption of CR onto Ce-BTC@MCC occurs as a monolayer on a homogeneous surface.

### Kinetic studies

To gain a deeper understanding of the adsorption mechanism and rate of CR adsorption onto Ce-BTC@MCC, it is crucial to investigate the adsorption kinetics. Common kinetic models were employed to analyze and fit the experimental data (Fig. 7b) and provide valuable insights into the adsorption kinetics. Lagergren’s pseudo-first-order model (Eq. (S6))^90^ and Ho and McKay’s pseudo-second-order model (Eq. (S7))^91^ were investigated, along with the Elovich model (Eq. (S8)) which is typically used for chemisorption^92^ (Fig. 7b). The kinetic parameters for the adsorption of CR onto Ce-BTC@MCC are summarized in Table 2. Among the three kinetic models investigated, the pseudo-second-order kinetic model exhibited the highest correlation coefficient (R² = 0.9878) and the lowest chi-square value (χ² = 27.2), indicating the best fit to the experimental data. Furthermore, the calculated q_e_ value from the pseudo-second-order fitted plot (206.1 mg/g) is closer to the experimental value (200 mg/g) than the pseudo-first-order model (179.8 mg/g). On the basis of the correlation coefficients and chi-square values presented in Table 2, the pseudo-second-order kinetic model provides the best fit to the experimental data and is therefore the most appropriate model to describe the adsorption kinetics of CR onto Ce-BTC@MCC.

The three previously studied models could not elucidate the diffusion mechanism of CR into Ce-BTC@MCC. Therefore, Weber and Morris’s intraparticle diffusion model was used instead (Eq. (S9))^93^. The intraparticle diffusion rate constant (*k*_*id*_) and boundary layer thickness (*C*) were calculated from the intraparticle diffusion plot (q_t_ vs. t^1/2^) (Table 2). A single linear plot passing through the origin is indicative of intraparticle diffusion as the sole rate-controlling step, whereas multiple linear portions suggest a more complex mechanism involving both intraparticle and boundary layer diffusion. The two-stage linear plot for CR adsorption onto Ce-BTC@MCC (Fig. 7c) suggests that both mechanisms are involved, with external and boundary diffusion dominating the first stage of high slope (35.1) and intraparticle diffusion dominating the second drop stage (4.2)^89,94^.

### Thermodynamic studies

To understand how temperature affects the adsorption process, we calculated thermodynamic parameters such as Gibbs free energy (∆G°), enthalpy (∆H°), and entropy (∆S°) using Van’t Hoff equations (Eqs. (S10-S12))^94–97^. The slope and intercept of the Van’t Hoff plot (ln Ke vs. 1/T) were used to determine ∆H° and ∆S° (see Fig. 7d; Table 3), according to Eqs. (S10-S12). The positive values of entropy and enthalpy, as presented in Table 3, confirm the endothermic nature of the adsorption process, in addition to a significant randomness at the solid-solution interface^94,97,98^. Typically, physical adsorption has a ∆G° between − 20 and 0 kJ/mol, whereas chemisorption ranges from − 80 to −400 kJ/mol^94,97,98^. The calculated ∆G° values (Table 3) for CR adsorption onto Ce-BTC@MCC fall within the range of physical adsorption. Additionally, the negative ∆G° values confirm that the adsorption process is spontaneously and physically driven^94,95,97,98^.

### Comparison with other adsorbents

Compared with other materials used to remove CR, Ce-BTC@MCC demonstrated superior adsorption capacity under optimal conditions (Table 4). This enhanced performance is likely due to the synergistic effect between Ce-BTC and MCC. In addition, strong interaction sites within the Ce-BTC@MCC matrix (e.g., hydrogen bonding, π-π stacking, and chelation) significantly enhance CR adsorption. Moreover, Ce-BTC@MCC is cost-effective as the estimated cost is significantly reduced when it is combined with MCC, which can be produced from recycled materials such as grass, bacteria, cottonwood, and agricultural waste, indicating its significant economic value^99,100^.

**Table 1:** Parameters of the adsorption isotherms for CR onto Ce-BTC@MCC.

Model	Parameters	Value of parameters
Langmuir	q_m_ (mg g^−1^)	926.3
	K_L_ (L mg^−1^)	0.0042
	R ^2^	0.9849
	χ ^2^	1909.2
Freundlish	K _F_	47.73
	N	2.6
	R ^2^	0.9571
	χ ^2^	4602.1
Temkin	K_T_ (L g^−1^)	0.11
	b_T_ (KJ mol^−1^)	151.44
	R ^2^	0.9460
	χ ^2^	5269.9
Redlich-Peterson	K_RP_ (L g^−1^)	5.45
	a_RP_ (L mg^−1^)	0.016
	G	0.87
	R ^2^	0.9771
	χ ^2^	2363.3
Hill	q_m_ (mg g^−1^)	1113.3
	K_H_ (mg L^−1^)	94.54
	N	0.75
	R ^2^	0.9805
	χ ^2^	2327.54

**Table 2:** Kinetic parameters and their correlation coefficients for the adsorption of CR onto Ce-BTC@MCC.

Model	Parameters	Value of parameters
Pseudo-first-order	*K*_***1***_ (min^−1^)	0.000032
	q_e_ (mg g^−1^)	179.8
	R ^2^	0.6046
	χ ^2^	456.45
Pseudo-second-order	*K*_***2***_ (g mg^−1^ min^−1^)	0.0048
	q_e_ (mg g^−1^)	206.1
	R ^2^	0.9878
	χ ^2^	27.2
Elovich	α (mg g^−1^ min^−1^)	2025.23
	β (g mg ^−1^ )	0.036
	R ^2^	0.9443
	χ ^2^	64.27
Intra-particle diffusion	*K*_*id,1*_ (mg g^−1^ min^−1/2^)	35.1
	*C*_*1*_ (mg g^−1^)	83.86
	R _1_^2^	0.9583
	*K*_*id,2*_ (mg g^−1^ min^−1/2^)	4.2
	*C*_2_ (mg g^−1^)	179.12
	R _2_^2^	0.6156

**Table 3:** Thermodynamic parameters for the adsorption of CR onto Ce-BTC@MCC.

Adsorbate	∆H ^◦^(kJ mol^−1^)	∆S^◦^(J mol^−1^ K^−1^)	∆G ^◦^(kJ mol^−1^)			
			298 K	308 K	318 K	328 K
CR	27.47	112.37	−5.45	−7.71	−9.95	−12.19

**Table 4:** Comparison of the adsorption capacity of the developed Ce-BTC@MCC composite for CR with other reported adsorbents.

Adsorbent	q_max_ (mg/g)	Reference
ZT-MOF@Ag@C	416.6	^101^
AlOOH/CoFe_2_O_4_	565.0	^102^
Fe_3_O_4_@bacteria	320.1	^103^
UiO-66-NH_2_@PEI	583.4	^104^
ZIF-8-PVA	829.4	^105^
ZIF-8@PDA	525.8	^106^
ATTM@ZIF-8	680.3	^107^
NH_2_-MIL-101(Fe)	248.4	^108^
Cu-MOF	119.8	^109^
CA-β-CD-MOF	900.9	^110^
IL@HBU-167	544.6	^111^
Fe_3_O_4_@TpPDA	179.4	^112^
Ni/Co-BTC hollow MOFs	168.1	^113^
Fe/MOF-5@CTS	219.8	^114^
Quasi-HKUST	715.0	^115^
Ce-BTC@MCC	926.3	This work

### Adsorption mechanism

The experimental results of CR adsorption aligned well with the Langmuir isotherm model with the highest R² value of 0.9849, demonstrating that the adsorption of CR onto occurs as a monolayer on a homogeneous surface. In addition, The R_L_ value for CR adsorption onto Ce-BTC@MCC was 0.073, close to zero, confirming favorable and irreversible adsorption. Thermodynamic studies have shown that the absorption of CR dye is spontaneous and physically driven. Furthermore, the pseudo-second-order model effectively explained the adsorption kinetics (R² = 0.9878, χ² = 27.2), as well as Weber-Morris intraparticle diffusion analysis indicates that CR adsorption process is controlled by both intraparticle and boundary layer diffusion mechanisms. The adsorption of CR onto the Ce-BTC@MCC adsorbent generally involves the following stages: migration to the sorbent surface, boundary layer diffusion, adsorption at surface active sites, intra-particle diffusion into pores, and interaction with external and internal active sites^13,116^.

**Scheme 2:**
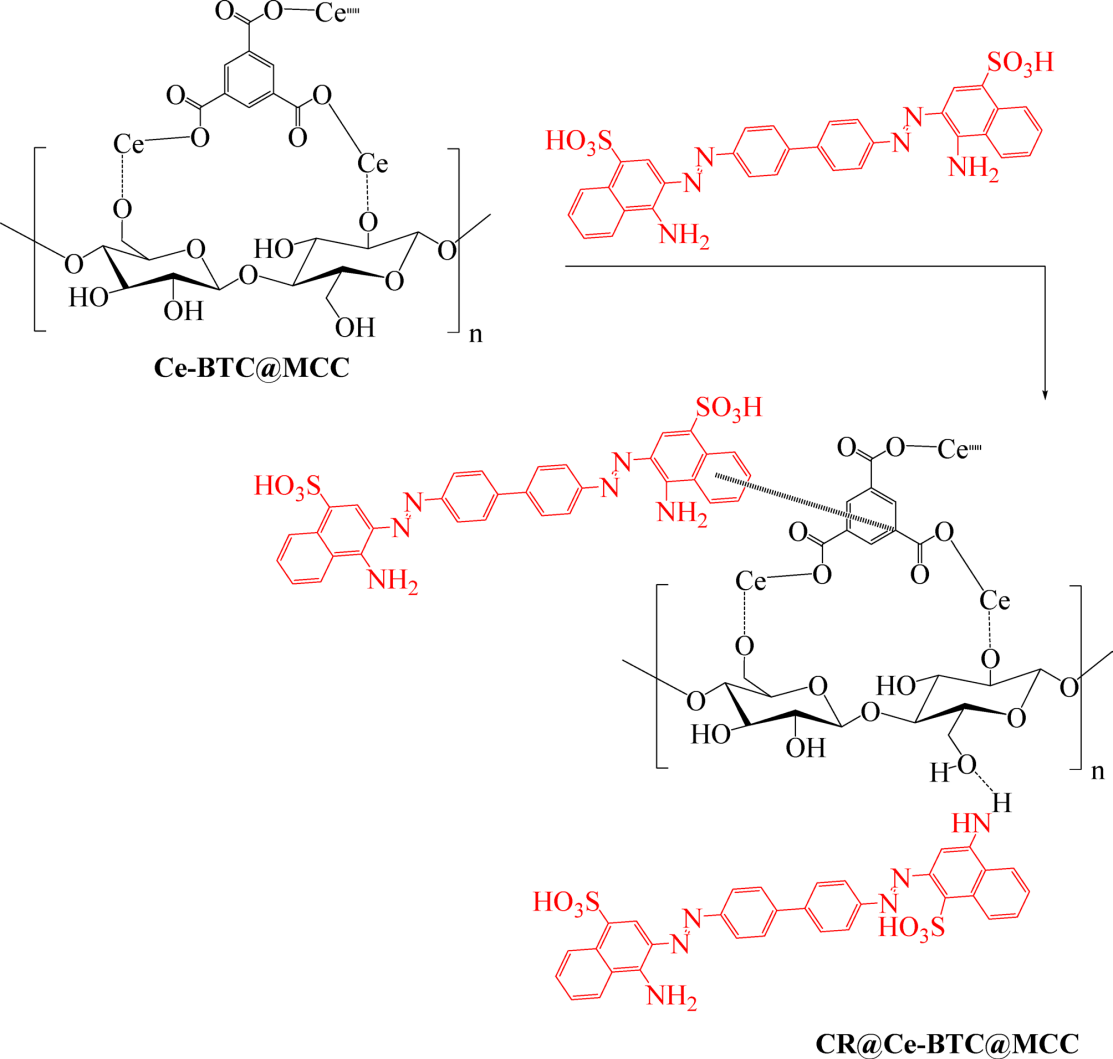
Adsorption mechanism of CR onto the Ce-BTC@MCC composite.

**Fig. 8:**
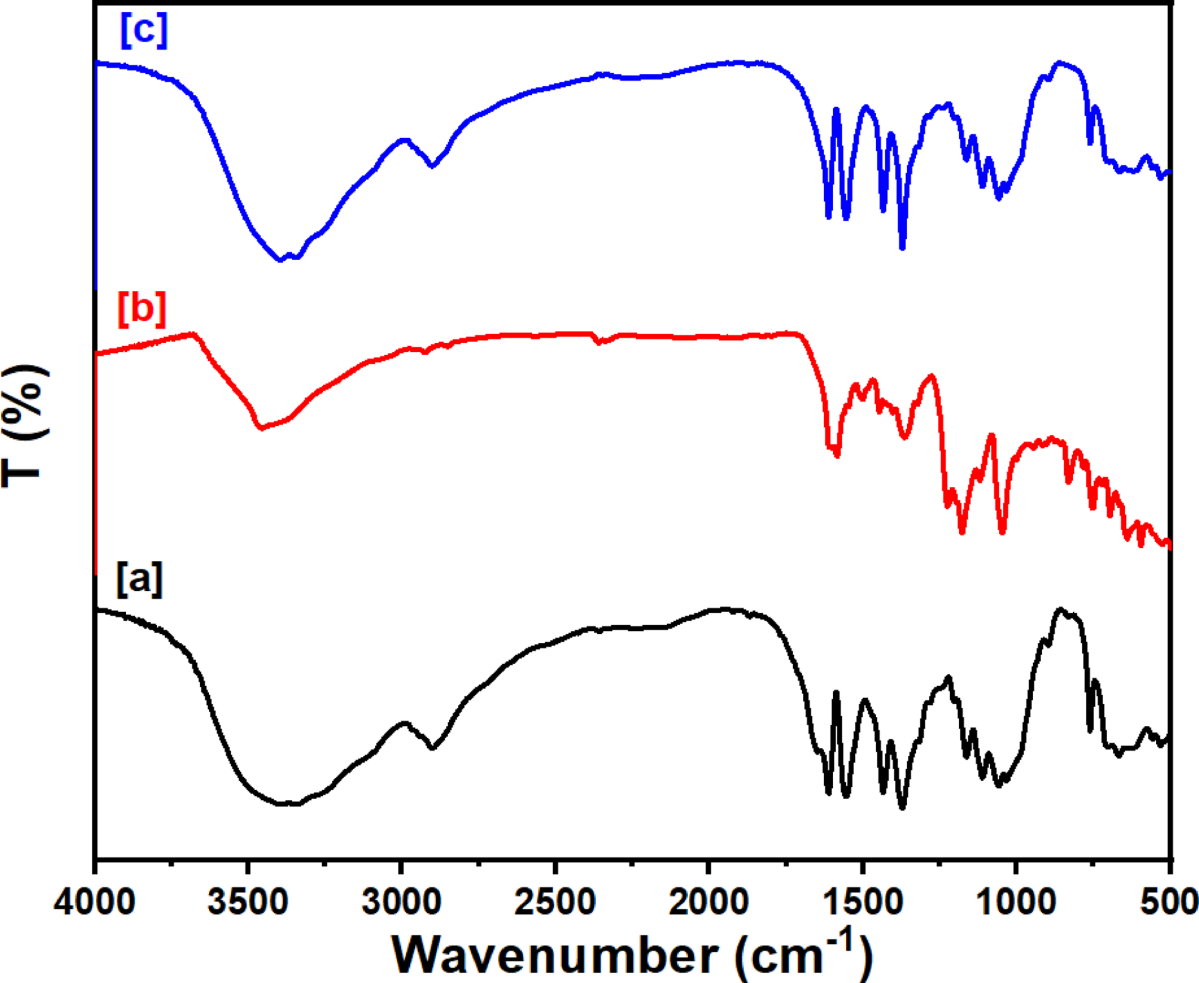
FTIR spectra of [a] Ce-BTC@MCC, [b] CR dye, and [c] CR adsorbed at Ce-BTC@MCC.

Investigations were conducted on the CR adsorption mechanism onto the Ce-BTC@MCC composite. Adsorption is significantly impacted by hydrogen bonding, chelation, π-π stacking interactions, and pore filling. As shown in Scheme 2, Ce-BTC@MCC is a mesoporous material that provides pore filling potential. The material can allow CR dye molecules to interact with the adsorbent if it contains enough mesopores. The CR dye molecules adsorb on the adsorbent more quickly because they can readily diffuse into the adsorbent and spread out into the pores and surfaces. Additionally, CR dyes exhibit strong interaction via the coordination between the nitrogen atoms of the -NH_2_ in CR and Ce cations on the surface of the composite. Another interaction affecting the adsorption properties is the hydrogen bonds formed between the delocalized π electron of the aromatic ring in the adsorbent and the nitrogen atoms of the -NH_2_ in the CR dye. Moreover, the numerous hydroxyl groups in MCC interact with CR molecules through hydrogen bonding interactions. In addition, the π-π stacking interactions occurred, because the CR molecule contains benzene rings and the prepared composite contains benzene rings. The adsorption process of CR dye molecules on the surface of the Ce-BTC@MCC composite is facilitated by the above interactions and their synergistic effects, as evidenced by the XPS analysis, and FTIR spectrum (Fig. 8).

### Selectivity study

**Fig. 9:**
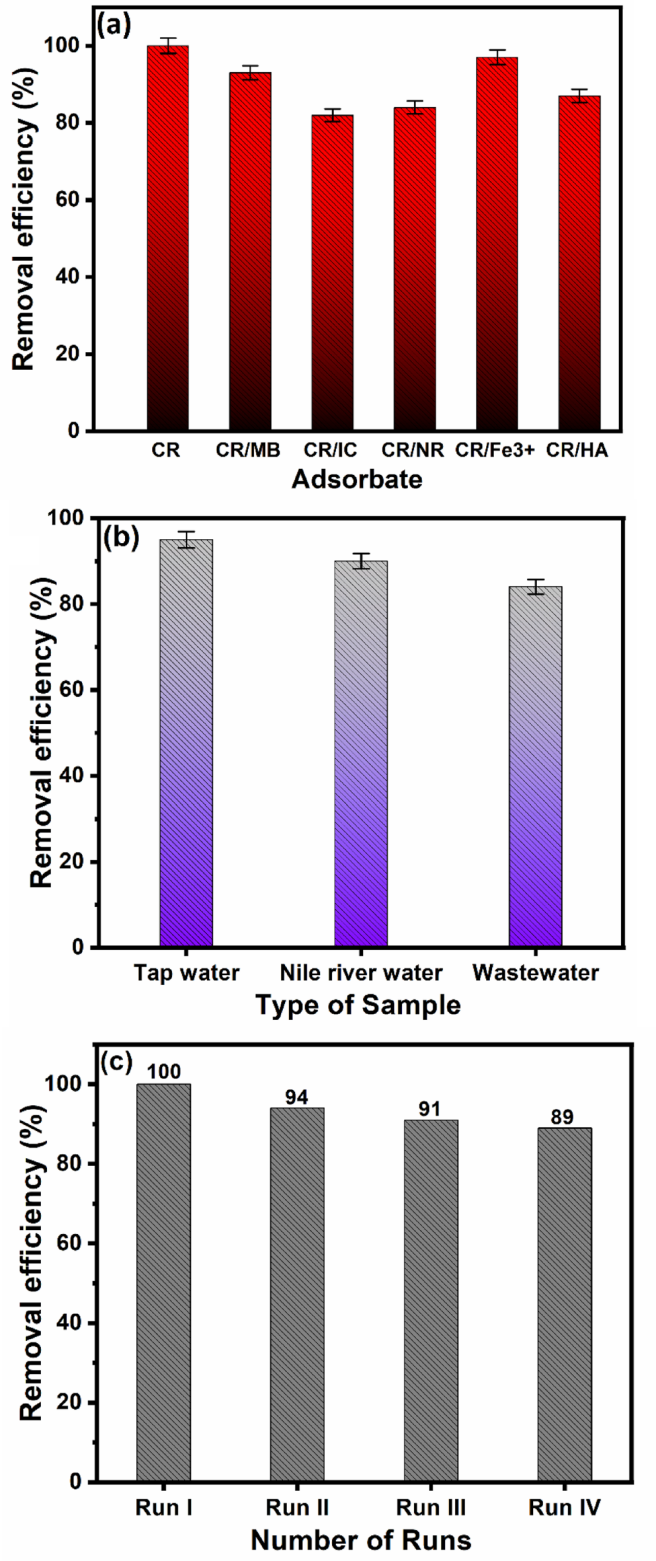
(a) Impact of interfering species on the adsorption of CR dye onto the Ce-BTC@MCC composite, (b) the efficiency of CR removal by Ce-BTC@MCC from various real water samples, and (c) the recyclability of Ce-BTC@MCC operated on CR dye (experimental conditions: pH = 5.0, contact time = 30 min, temperature = 298 K, concentration of CR = 100 mg/L, and adsorbent dose = 0.4 g/L).

It was crucial to assess the adsorption behavior of the created Ce-BTC@MCC adsorbent toward CR dye in the presence of the interfering cationic Methylene Blue (MB), anionic Indigo Carmine (IC), Neutral Red (NR) dyes, Fe^3+^ ions as well as Humic acid (HA) in binary solutions as shown in Fig. 9 (a). The test was administered in the following manner: 0.02 g of Ce-BTC@MCC was added to 50 mL of single-pollutant or multipollutant systems (100 mg/L). The mixtures were subsequently centrifuged, and a spectrophotometer was used to measure the quantity of CR dye that persisted. Among the various types of interfering substances, the produced Ce-BTC@MCC adsorbent showed significant selectivity for CR dye and removed it with great efficacy. It is noteworthy that Ce-BTC@MCC demonstrates exceptional effectiveness in adsorbing CR dye, achieving removal efficiencies of approximately 100% in binary solution systems.

### Application study

To demonstrate the practicality and analytical effectiveness of Ce-BTC@MCC, various environmental water samples, such as tap water, Nile River water, and wastewater, were analyzed. The samples were examined without any prior treatment for the removal of organic substances or other elements. The amount of CR dye was analyzed in the three samples and discovered to be below the detection limit of UV–vis. The three actual samples were treated with a dose of 100 mg/L CR dye along with 0.02 g Ce-BTC@MCC at pH 5, and the mixture was stirred for 30 min at 298 K. As illustrated in Fig. 9(b), the percentages of removal efficiency were measured to be 95%, 90%, and 84% for tap water, Nile River water, and wastewater, respectively. These findings demonstrate that Ce-BTC@MCC has great potential as a highly efficient absorbent for eliminating CR from actual aqueous samples.

### Reusability test

The recyclability test fundamentally acts as a benchmark that should be used to prove the feasibility of any suggested adsorption research. To assess whether the produced Ce-BTC@MCC could be reused, a more extensive study was conducted over four adsorption/desorption cycles. The adsorbent was first soaked in distilled water containing a few drops of 0.1 M ammonia, which served to adjust the pH to approximately 8–9, creating optimal conditions for dye desorption. Gentle stirring was applied for 2 h to facilitate the removal of the Congo red dye molecules from the active sites of the Ce-BTC@MCC composite. Then, the adsorbent was thoroughly rinsed with distilled water to remove any residual ammonia and dye solution. The desorption process was further enhanced by immersing the adsorbent in ethyl alcohol, which helped to further disrupt the interactions between the dye and the adsorbent surface. Finally, the regenerated adsorbent was dried at 60 °C for 24 h in an oven to restore its original structural integrity and prepare it for subsequent adsorption cycles. Figure 9(c) shows that following the fourth cycle, the removal percentage of CR decreased by only approximately 10%, indicating the remarkable durability and reusability of the fabricated Ce-BTC@MCC adsorbent.

## Conclusion

This research shows significant advancements in adsorption materials through the fabrication of a novel composite, Ce-BTC@MCC, which integrates low-cost microcrystalline cellulose (MCC). FTIR, XRD, BET, and SEM/EDX techniques were used to analyze the surface features and chemical framework of the Ce-BTC@MCC composite thoroughly. The primary goal of this research was to investigate the capacity of the newly developed Ce-BTC@MCC composite to adsorb Congo red (CR) dye from aqueous solutions. The optimal 20 wt% Ce-BTC content was determined through a series of adsorption experiments, where the adsorption capacities were measured at varying Ce-BTC loadings. The resulting composites showed better removal effectiveness compared to their individual components, emphasizing the synergistic impact of the Ce-BTC MOF and MCC. Factors such as pH, contact time, initial concentration, and temperature significantly affect the adsorption process. The experimental results of CR adsorption aligned well with the Langmuir isotherm model with the highest R² value of 0.9849 and lowest χ² value of 1909.2, demonstrating that the adsorption of CR onto Ce-BTC@MCC occurs as a monolayer on a homogeneous surface. The Ce-BTC@MCC composite, under optimized conditions, showed a maximum adsorption capacity of 926.3 mg/g for CR dye, surpassing that of most previous adsorbents. In addition, the pseudo-second-order model effectively explained the adsorption kinetics, exhibited the highest correlation coefficient (R² = 0.9878) and the lowest chi-square value (χ² = 27.2). Weber-Morris intraparticle diffusion analysis indicates that the CR adsorption process is controlled by both intraparticle and boundary layer diffusion mechanisms. Thermodynamic research has shown that the absorption of dye is spontaneous and physically driven. The findings also show that the Ce-BTC@MCC composite can effectively adsorb CR dye from real water samples with high selectivity, resulting in great durability and reusability. This study also explored a credible adsorption mechanism for CR onto Ce-BTC@MCC. Accordingly, Ce-BTC@MCC composite is an eco-friendly and efficient adsorbent for eliminating CR dye from different types of water samples. This study advances adsorption materials and provides a promising avenue for tackling water pollution issues with implications for environmental sustainability.

## Electronic supplementary material

Below is the link to the electronic supplementary material.

Supplementary Material 1

## Data Availability

All data generated or analysed during this study are included in this published article and its supplementary information files.
