# Supplementary material for: Efficient adsorptive removal of hazardous congo red dye using Ce-BTC@microcrystalline cellulose composite
Source: Sci Rep. 2025 Jun 5;15:19734. doi: 10.1038/s41598-025-04085-2 (PMC12141572; doi:10.1038/s41598-025-04085-2)
Supplement: Supplementary file 1 — Supplementary Material 1 [file 41598_2025_4085_MOESM1_ESM.docx]

**Supporting information for:**

**Efficient Adsorptive Removal of Hazardous Congo Red Dye Using**

**Ce-BTC@Microcrystalline Cellulose Composite**

Mostafa A. Sayed^1^, Reda M. Abdelhameed^2^, Ibrahim H. A. Badr^1,3^*, Ali M. Abdel-Aziz^1^

^1^Chemistry Department, Faculty of Science, Ain-Shams University, Cairo 11566, Egypt

^2^Applied Organic Chemistry Department, [National Research Centre](https://scholar.google.com/citations?view_op=view_org&hl=en&org=4287808777764934601), Dokki, Giza, Egypt

^3^Department of Chemistry, Faculty of Science, Galala University, New Galala City, Suez, Egypt

* E-mail : [ihbadr@sci.asu.edu.eg](mailto:ihbadr@sci.asu.edu.eg), [ibrahim.badr@gu.edu.eg](mailto:ibrahim.badr@gu.edu.eg)

**Instrumentation**

SEM images were obtained by an HRSEM Quanta FEG 250 with field emission gun to analyze the morphological properties of MOFs. A Malvern Panalytical X'PertPRO PANalytical diffractometer (K X-ray at 45 kV, 40 mA, = 1.5406) was used to characterize both crystallinity and phase purity of the prepared MOFs. The Fourier transforms infrared spectra of MOFs were measured using a JASCO FT/IR 6100 spectrometer. BET measurements were carried out using Autosorb-01(Quantachrome TouchWin™).

**Theories of adsorption isotherm, kinetics, and thermodynamic studies**

***Adsorption isotherm models***

The obtained equilibrium data for MB and Sm(III) onto MIL-68-NH_2_@MCC were modeled with the Langmuir (Eq. (S1)), Freundlich (Eq. (S2)), Temkin (Eq. (S3)), Redlich-Peterson (Eq. (S4)), and Hill (Eq. (S5)) isotherm models. The nonlinear forms of the five isotherms are:

$$\mathbf{q}_{\mathbf{e}}\mathbf{=}\frac{\mathbf{q}_{\mathbf{m}} \mathbf{K}_{\mathbf{L}} \mathbf{C}_{\mathbf{e}}}{\mathbf{1+}\mathbf{K}_{\mathbf{L}} \mathbf{C}_{\mathbf{e}}} \mathbf{Eq. (S1)}$$

$$\mathbf{q}_{\mathbf{e}}\mathbf{=}\mathbf{K}_{\mathbf{F}} \mathbf{C}_{\mathbf{e}}^{\mathbf{1/n}} \mathbf{Eq. (S2)}$$

$$\mathbf{q}_{\mathbf{e}}\mathbf{=}\frac{\mathbf{RT}}{\mathbf{b}_{\mathbf{T}}}\mathbf{ln}\mathbf{K}_{\mathbf{T}}\mathbf{C}_{\mathbf{e}} \mathbf{Eq. (S3)}$$

$$\mathbf{q}_{\mathbf{e}}\mathbf{=}\frac{\mathbf{K}_{\mathbf{RP}} \mathbf{C}_{\mathbf{e}}}{\mathbf{1+}\mathbf{a}_{\mathbf{RP}} \mathbf{C}_{\mathbf{e}}^{\mathbf{g}}} \mathbf{Eq. (S4)}$$

$$\mathbf{q}_{\mathbf{e}}\mathbf{=}\mathbf{q}_{\mathbf{m}}\frac{\mathbf{C}_{\mathbf{e}}^{\mathbf{n}}}{\mathbf{K}_{\mathbf{H}}\mathbf{+}\mathbf{C}_{\mathbf{e}}^{\mathbf{n}}} \mathbf{Eq. (S5)}$$

where C_e_ (mg/L) and q_e_ (mg/g) are the equilibrium concentration, and the amount of MB or Sm(III) adsorbed at equilibrium, respectively. K_L_ (L/mg) and q_m_ (mg/g) are Langmuir constant, and the monolayer adsorption capacity, respectively. K_F_ and n are the Freundlich constants, which are indicators of adsorption capacity and adsorption intensity, respectively.

Temkin constant (b_T_) is related to the heat of adsorption (kJ/mol), K_T_ (L/g) is the Temkin equilibrium binding constant. K_RP_ (L/g) and a_RP_ (L/mg) are the Redlich-Peterson constants; g is an exponent whose value must lie between 0 and 1. K_H_ (mg/L) is the Hill constant that represents the concentration for which q_e_ is equal to 0.5 q_m_, and n (dimensionless) is the Hill coefficient of sigmoidicity which is related to the degree of cooperation between binding sites.

***Kinetics models***

Lagergren’s pseudo-first-order (Eq. (S6)), Ho and McKay’s pseudo-second-order (Eq. (S7)), and the Elovich model (Eq. (S8)) adsorption kinetics models were used for fitting the kinetic adsorption data. The mechanism of adsorption onto MIL-68-NH_2_@MCC was investigated using Weber and Morris’s intraparticle diffusion (Eq. (S9)):

$$\mathbf{q}_{\mathbf{t}}\mathbf{=}\mathbf{q}_{\mathbf{e}} \left( \mathbf{1-}\mathbf{e}^{\mathbf{-}\boldsymbol{K}_{\mathbf{1}}\mathbf{t}} \right) \mathbf{Eq. (S6)}$$

$$\mathbf{q}_{\mathbf{t}}\mathbf{=}\frac{\mathbf{q}_{\mathbf{e}}^{\mathbf{2}} \boldsymbol{K}_{\mathbf{2}}\mathbf{t}}{\mathbf{1+}\boldsymbol{K}_{\mathbf{2}}\mathbf{q}_{\mathbf{e}}\mathbf{t}} \mathbf{Eq. (S7)}$$

$$\mathbf{q}_{\mathbf{t}}\mathbf{=}\frac{\mathbf{1}}{\boldsymbol{\beta}}\mathbf{ln}\left( \boldsymbol{1+ \alpha\beta t} \right) \mathbf{Eq. (S8)}$$

$$\mathbf{q}_{\mathbf{t}}\mathbf{=}\boldsymbol{K}_{\boldsymbol{id}}\mathbf{t}^{\mathbf{1}/\mathbf{2}}\mathbf{+}\boldsymbol{C}_{\boldsymbol{i}} \mathbf{Eq. (S9)}$$

where q_e_ (mg/g) and q_t_ (mg/g) are the amounts adsorbed at equilibrium and at time t, respectively. *k_1_* (min^−1^) and *k_2_* (g mg^−1^ min^-1^) are the pseudo-first order and pseudo-second order adsorption rate constants, respectively. *k_i_*_d_ (mg g^-1^ min^-1/2^) is the intraparticle diffusion rate constant, and *C* is a constant related to the diffusion resistance. α (mg/g.min) is the initial rate constant and β (g/mg) is the desorption constant.

***Thermodynamic studies***

To assess the effect of temperature on the nature of the studied adsorption processes, thermodynamic parameters including the Gibbs free energy (**∆*G*^◦^**), standard enthalpy (**∆*H*^◦^**), and entropy (**∆*S*^◦^**) were determined using the following Van’t Hoff equations:

$$\mathbf{ln}\boldsymbol{K}_{\boldsymbol{e}}\mathbf{=}\frac{\boldsymbol{\Delta}\boldsymbol{S}^{\boldsymbol{^{\circ}}}}{\boldsymbol{R}}\mathbf{-}\frac{\boldsymbol{\Delta}\boldsymbol{H}^{\boldsymbol{^{\circ}}}}{\boldsymbol{RT}}\mathbf{Eq.(S10)}$$

$$\boldsymbol{\Delta}\boldsymbol{G}^{\boldsymbol{^{\circ}}}\mathbf{=}\boldsymbol{\Delta}\boldsymbol{H}^{\boldsymbol{^{\circ}}}\mathbf{-}\boldsymbol{T}\boldsymbol{\Delta}\boldsymbol{S}^{\boldsymbol{^{\circ}}} \mathbf{Eq. (S11)}$$

$$\boldsymbol{K}_{\mathbf{e}}\mathbf{=}\frac{\boldsymbol{q}_{\boldsymbol{e}}}{\boldsymbol{C}_{\boldsymbol{e}}} \mathbf{Eq.(S12)}$$

Where *K*_e_ is the thermodynamic equilibrium constant, *q*_e_ is the amount of adsorbate adsorbed from the solution at equilibrium (mg/L), *C*_e_ is the equilibrium concentration of adsorbate in the solution (mg/L), R is the universal gas constant (8.314 J mol^-1^ K^-1^), and T is the temperature in (K).

**Fig. S1.** XPS spectrum of Ce-BTC@MCC with their corresponding elements (a) survey scan, (b) C 1 s, (c) Ce 3d and (d) O 1 s.

**Fig. S2.** XPS spectrum of Ce-BTC@MCC after adsorption with their corresponding elements (a) survey scan, (b) C 1 s, (c) Ce 3d and (d) O 1s.


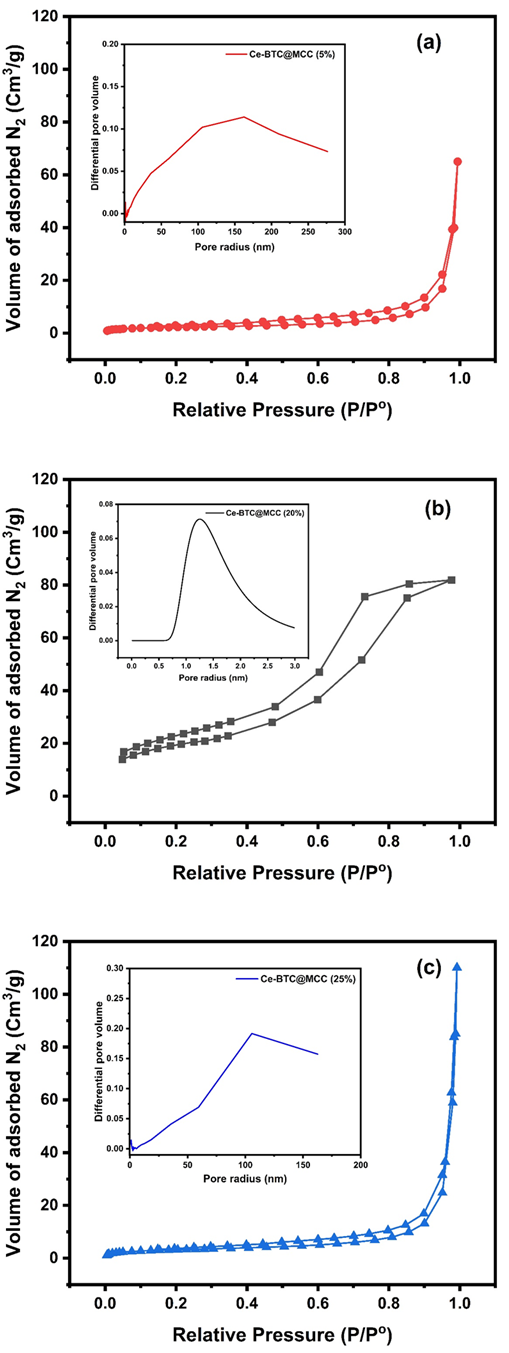


**Fig. S3**. N_2_-adsoption/desorption isotherms and pore size distributions (inset) of (a) 5 wt% Ce-BTC@MCC, (b) 20 wt% Ce-BTC@MCC, and (c) 25 wt% Ce-BTC@MCC composites.


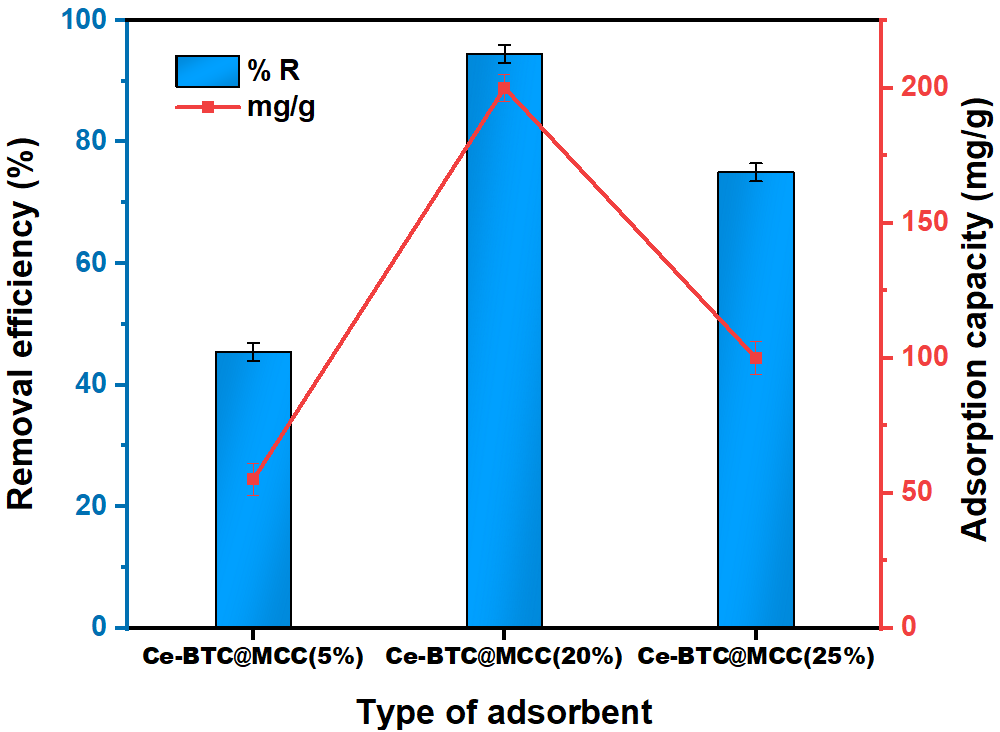


**Fig. S4.** Adsorption efficiency of the material at varying Ce-BTC:MCC ratios (experimental conditions: pH = 5.0, contact time = 30 minutes, Temperature = 298 K, concentration of CR = 100 mg/L, and adsorbent dose = 0.4 g/L).

**Fig. S5.** Point of zero charge (pHpzc) of Ce-BTC@MCC composite.
